# Construction and validation of an endoscopic ultrasonography-based ultrasomics nomogram for differentiating pancreatic neuroendocrine tumors from pancreatic cancer

**DOI:** 10.3389/fonc.2024.1359364

**Published:** 2024-05-23

**Authors:** Shuangyang Mo, Cheng Huang, Yingwei Wang, Huaying Zhao, Haixiao Wei, Haiyan Qin, Haixing Jiang, Shanyu Qin

**Affiliations:** ^1^ Gastroenterology Department, Liuzhou People’s Hospital Affiliated to Guangxi Medical University, Liuzhou, China; ^2^ Gastroenterology Department, The First Affiliated Hospital of Guangxi Medical University, Nanning, China; ^3^ Oncology Department, Liuzhou People’s Hospital Affiliated to Guangxi Medical University, Liuzhou, China

**Keywords:** pancreatic neuroendocrine tumors, pancreatic cancer, endoscopic ultrasonography, ultrasomics, machine learning, nomogram

## Abstract

**Objectives:**

To develop and validate various ultrasomics models based on endoscopic ultrasonography (EUS) for retrospective differentiating pancreatic neuroendocrine tumors (PNET) from pancreatic cancer.

**Methods:**

A total of 231 patients, comprising 127 with pancreatic cancer and 104 with PNET, were retrospectively enrolled. These patients were randomly divided into either a training or test cohort at a ratio of 7:3. Ultrasomics features were extracted from conventional EUS images, focusing on delineating the region of interest (ROI) for pancreatic lesions. Subsequently, dimensionality reduction of the ultrasomics features was performed by applying the Mann-Whitney test and least absolute shrinkage and selection operator (LASSO) algorithm. Eight machine learning algorithms, namely logistic regression (LR), light gradient boosting machine (LightGBM), multilayer perceptron (MLP), random forest (RF), extra trees, k nearest neighbors (KNN), support vector machine (SVM), and extreme gradient boosting (XGBoost), were employed to train prediction models using nonzero coefficient features. The optimal ultrasomics model was determined using a ROC curve and utilized for subsequent analysis. Clinical-ultrasonic features were assessed using both univariate and multivariate logistic regression. An ultrasomics nomogram model, integrating both ultrasomics and clinical-ultrasonic features, was developed.

**Results:**

A total of 107 EUS-based ultrasomics features were extracted, and 6 features with nonzero coefficients were ultimately retained. Among the eight ultrasomics models based on machine learning algorithms, the RF model exhibited superior performance with an AUC= 0.999 (95% CI 0.9977 - 1.0000) in the training cohort and an AUC= 0.649 (95% CI 0.5215 - 0.7760) in the test cohort. A clinical-ultrasonic model was established and evaluated, yielding an AUC of 0.999 (95% CI 0.9961 - 1.0000) in the training cohort and 0.847 (95% CI 0.7543 - 0.9391) in the test cohort. Subsequently, the ultrasomics nomogram demonstrated a significant improvement in prediction accuracy in the test cohort, as evidenced by an AUC of 0.884 (95% CI 0.8047 - 0.9635) and confirmed by the Delong test. The calibration curve and decision curve analysis (DCA) depicted this ultrasomics nomogram demonstrated superior accuracy. They also yielded the highest net benefit for clinical decision-making compared to alternative models.

**Conclusions:**

A novel ultrasomics nomogram was proposed and validated, that integrated clinical-ultrasonic and ultrasomics features obtained through EUS, aiming to accurately and efficiently identify pancreatic cancer and PNET.

## Introduction

Pancreatic neuroendocrine tumors (PNET) are a rare occurrence, constituting approximately 3%-5% of all pancreatic tumors, and exhibit a significant degree of heterogeneity (1). These tumors originate from pancreatic endocrine tissues and rank as the second most prevalent pancreatic tumor, surpassed only by pancreatic cancer (2). PNET are characterized by extremely variable biological features spanning from low-grade malignant tumors to immensely aggressive ones (3). Based on the capability to secrete biologically active hormones and characteristic clinical symptoms, PNET can be categorized into two distinct categories: namely functional (F-PNET) and non-functional (NF-PNET) (4). NF-PNET exhibit a greater incidence and a more unfavorable prognosis than F-PNET, except the insulinomas (5). The preoperative identification of PNET poses a considerable challenge, relying primarily on pathological examination and immunohistochemistry, with pancreatic cancer being the most critical differential diagnosis (6). Consequently, the prompt and accurate diagnosis and treatment of PNET hold utmost significance (7).

Currently, a range of diagnostic imaging modalities, such as computed tomography (CT), magnetic resonance imaging (MRI), and transabdominal ultrasonography (US) are commonly applied for the diagnosis of PNET. Compared to CT and MRI, endoscopic ultrasonography (EUS) is regarded as one of the most accurate imaging modalities for the diagnosis of pancreatic diseases because of its ability to provide high-definition images of the pancreas and its sensitivity ranging from 57% to 94% (8). According to the consensus guidelines of the European Neuroendocrine Tumor Society (ENETS) in 2023, EUS is considered the preferred imaging modality following negative findings from alternative noninvasive imaging techniques. This preference is due to EUS’s ability to offer meticulous observation and estimation of PNET, as well as its capacity to conduct a comprehensive scan of the whole pancreas (9).

Currently, ongoing advancements in computer-aided detection (CAD) and artificial intelligence (AI) have contributed to the gradual emergence of radiomics as a promising research domain. Radiomics enables the extraction and analysis of numerous objective and internal image features through high-throughput techniques. These features are subsequently utilized to develop diverse tumor diagnosis and prediction models using various machine learning, deep learning, and other algorithmic approaches (10). Numerous studies have elucidated the utility of CT-based and MRI-based radiomics in diagnosing and predicting PNET, showcasing its efficacy (11, 12). However, MRI is contraindicated for certain populations, including individuals with claustrophobia or metal implants. Additionally, the time-consuming and costly nature of MRI restricts its widespread clinical utility (13) Furthermore, CT presents drawbacks such as radiation exposure risks and potential harm from contrast agents (14).

Ultrasomics, a subfield of radiomics, involves the conversion of digitally encrypted medical images containing tumor pathophysiology information into high-dimensional data that can be analyzed. This technique has demonstrated efficacy in the precise diagnosis of a range of malignant tumors, including liver cancer, thyroid cancer, and breast cancer, yielding favorable outcomes (15). However, the efficiency of ultrasomics based on normal EUS images in enhancing the clinical diagnostic efficiency of PNET, despite EUS being acknowledged as an exceptional imaging modality, has not been validated.

In this research, a range of prevalent machine learning algorithms was employed to develop and assess an effective combined nomogram model based on ultrasomics and clinical signatures, to distinguish PNET from pancreatic cancer.

## Materials and methods

### Patient population

This retrospective study at our institution was approved by the institutional ethics review board, which waived the requirement for patient approval or signed informed consent to review medical images or information. A total of 231 patients underwent pancreatic surgery operation or EUS-guided fine-needle aspiration (EUS-FNA) at our institution (The First Affiliated Hospital of Guangxi Medical University) between January 2013 and October 2023, comprising 127 with pancreatic cancer and 104 with PNET, were retrospectively enrolled. The inclusion and exclusion criteria are outlined as follows.

**Figure 1:**
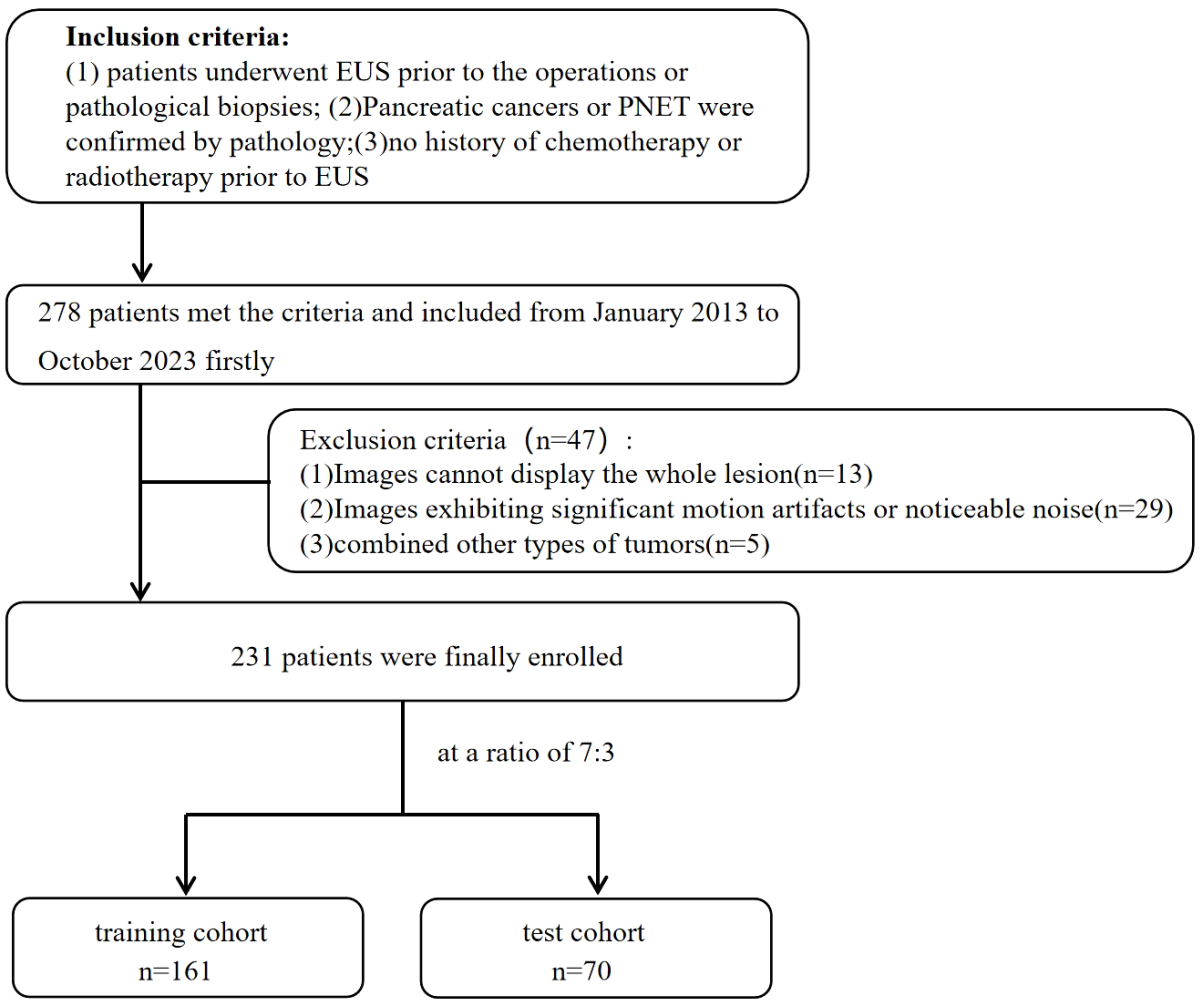
Flowchart for enrolling the study population.

The inclusion criteria for patients were as follows: (1) underwent preoperative EUS scan of the pancreas meticulously; (2) had pancreatic cancers or PNET confirmed by postoperative pathology or EUS-FNA pathology; and (3) had complete and clear EUS images available before the patient’s preoperative or pathological biopsies. (4) Patients who received no chemotherapy or radiotherapy before EUS. The exclusion criteria for patients were as follows:(1) inability to display the whole lesion; (2) significant motion artifacts or noticeable noise; (3) presence of other types of tumors. The enrolled patients were randomized into a training cohort and a test cohort at a ratio of 7:3. The flowchart for enrolling the study population is shown in **Figure 1**.

### EUS image acquisition

The study employed the standard dynamic EUS scan procedure using the EU-ME2 (Olympus, Japan) and SU-9000 (FUJIFILM, Japan) devices. A highly experienced EUS specialist with a record of more than 5000 EUS procedures meticulously scanned the entire pancreatic region and obtained high-resolution images of the pancreatic masses. At the same time, to reduce the image bias caused by different devices as much as possible, these images were consistently standardized with a window width of 250 Hounsfield units (HU) and a window level of 125 HU. The imaging records were gathered by retrieving data from our institutional Picture Archive and Communication System (PACS).

### Ultrasonic and clinical data analysis

This study retrospectively analyzed multiple clinical parameters, such as age, gender, and pathological diagnosis. To enhance the study’s credibility, all EUS images were meticulously examined and evaluated by two proficient UES experts, each with 6–7 years of experience in pancreatic EUS. In the case of disagreement, a consensus would be reached by consultation. Crucially, these experts were blinded to the histopathological and clinical data about the analyzed cases.

This study examined various parameters and features of the pancreatic masses, including its location, echo characteristics, uniformity of the echo, maximum diameter, shape, margin characteristics, and the presence of calcifications or cystic degeneration, via EUS. A detailed explanation of the ultrasonic features can be found in the **Supplementary Material**. In instances where multiple lesions were present in the pancreas, the analysis primarily concentrated on the largest lesion with confirmed pathology.

### Image segmentation

**Figure 2:**
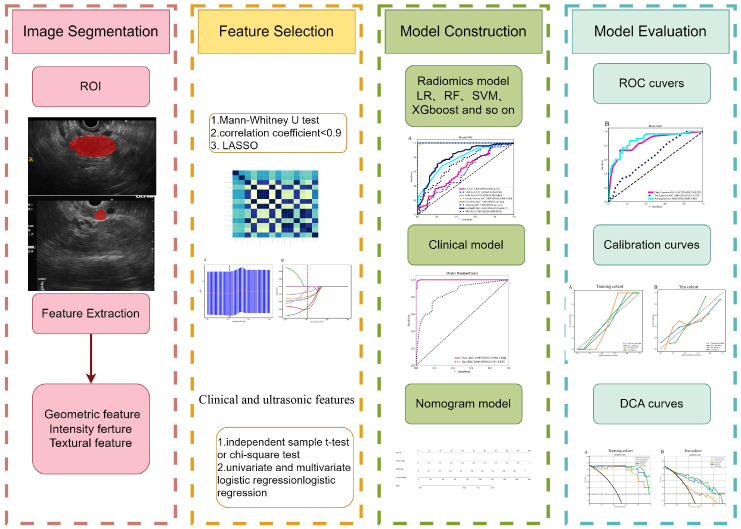
The workflow for this study.

The images were stored in Digital Imaging and Communications in Medicine (DICOM) format. Two EUS specialists, each with 6 and 7 years of experience, manually delineated the region of interest (ROI) using a gray-scale EUS image of the most extensive long-axis cross-section layer for pancreatic masses. This segmentation was performed utilizing ITK-SNAP software (version 3.8.1), accessed at http://www.itksnap.org. The specialists were unaware of the patients’ histopathological diagnosis. The lesions were captured carefully along the margins on conventional EUS images, excluding adjacent normal tissue, vessels, bile ducts, and pancreatic ducts. A comprehensive diagram can be found in **Figure 2**.

Standardization techniques were employed to preprocess the images and data, thereby guaranteeing the replicability of the results. Both intra-observer and inter-observer replicability were assessed using the intra-class correlation coefficient (ICC). A group of 102 patients, comprising 81 individuals with pancreatic cancer and 21 with PNET, was randomly chosen, and following a two-week interval, the same EUS specialists performed ROI segmentation once more. An ICC value exceeding 0.9 indicated a remarkable level of agreement.

### Ultrasomics feature extraction

The classification of handcrafted features can be divided into three distinct categories: geometric, intensity, and textural. Geometric features pertain to the three-dimensional morphological attributes of tumors. The intensity features encompass the statistical distribution of voxel intensities within the tumor in the first order. Conversely, textural features describe patterns and higher-order spatial distributions of intensities. This article utilized a range of techniques, including Gray Level Co-occurrence Matrix (GLCM), Gray Level Run Length Matrix (GLRLM), Gray Level Size Zone Matrix (GLSZM), and Neighborhood Gray-level Difference Matrix (NGTDM), to extract texture features. The algorithms employed to extract radiomic features were based on the image biomarker standardization initiative (IBSI) (16).

### Ultrasomics feature selection

To ascertain the dependability of the ultrasomics features, we conducted a Mann−Whitney U test to compare the PNET and pancreatic cancer cohorts, followed by feature screening. Subsequently, only ultrasomics features exhibiting *p*<0.05 were retained for subsequent analysis.

Correlation coefficient screening was conducted utilizing Spearman’s rank correlation coefficient to assess the interrelationship between each ultrasomics feature, aiming to ensure the robustness of the features (**Supplementary Figure 1**). Any feature with a correlation coefficient greater than 0.9 between any two features was retained with one of them. A greedy recursive deletion method was employed for feature filtration to enhance feature representation, systematically removing the feature with the highest redundancy within the existing set. As a result, a total of 8 ultrasomics features were ultimately preserved.

Finally, the 10-fold cross-validation method was used to identify ultrasomics features with nonzero coefficients using the least absolute shrinkage and selection operator (LASSO) regression model. All ultrasomics feature selection procedures were conducted within the training cohort and subsequently applied to the test cohort. Ultrasomic features with non-zero coefficients were selected for inclusion in the regression model and combined to form an ultrasomics signature. Subsequently, each patient was assigned an ultrasomics score by applying a linear combination of the selected ultrasomics features and their corresponding coefficients. LASSO regression analysis was conducted utilizing the Python scikit-learn package.

### Construction of ultrasomics signature model

The ultimate retained ultrasomics features were utilized in the development of ultrasomics models. To identify a classifier model with optimal tumor data recognition, our study employed eight prominent machine learning algorithms training models, including logistic regression (LR), light gradient boosting machine (LightGBM), multilayer perceptron (MLP), random forest (RF), extra trees, k nearest neighbors (KNN), support vector machine (SVM), and extreme gradient boosting (XGBoost). The definitive ultrasomics models were obtained through 5-fold cross-validation. The diagnostic efficacy of these different machine learning models was assessed through the evaluation of metrics such as the area under the receiver operating characteristic curve (AUC), sensitivity, accuracy, specificity, positive predictive value (PPV), and negative predictive value (NPV). Ultimately, the most optimal ultrasomics model was chosen and defined as the ultrasomics signature model.

### Clinical- ultrasonic signature model

Furthermore, we conducted univariate logistic regression analysis on each clinical predictor variable, encompassing both clinical and ultrasonic characteristics. To determine statistically significant clinical-ultrasonic features and establish the clinical-ultrasonic signature model, we performed a multivariate logistic regression. This allowed us to calculate the odds ratio (OR) and 95% confidence interval (CI) for each variable.

The identical ultrasomics signature model was performed to construct the clinical-ultrasonic signature model through the same machine learning algorithm. To ensure equitable comparison, a fixed 5-fold cross-validation and test cohort were utilized. The performance of this clinical-ultrasonic signature model was evaluated using various metrics such as the area under the curve (AUC), accuracy, sensitivity, specificity, PPV, and NPV. Additionally, the net benefit of the clinical-ultrasonic signature model in identifying pancreatic cancer and PNET was quantified through the use of decision curve analysis (DCA), which involved the application of diverse threshold probabilities in both the training and test cohorts.

### Ultrasomics nomogram establishment and assessment

Finally, an ultrasomics nomogram was developed in the test cohort to intuitively and efficiently evaluate the incremental predictive value of the ultrasomics signature for the clinical-ultrasonic signature. Based on logistic regression analysis, the ultrasomics nomogram was constructed by integrating the ultrasomics signature with the clinical-ultrasonic signature. The consistency between the prediction of the nomogram and the actual observation was compared by calculating the calibration curve, which compared the prediction of the nomogram with the actual observation.

The diagnostic effectiveness of the ultrasomics nomogram was evaluated in both the training and test cohorts by constructing receiver operating characteristic (ROC) curves. Subsequently, the Delong test was conducted to compare the performance of the ultrasomics nomogram, clinical-ultrasonic signature model, and ultrasomics signature model in terms of the AUC.

The calibration efficiency of the ultrasomics nomogram was assessed by constructing calibration curves. At the same time, the Hosmer-Lemeshow (H-L) analytical fit was employed to evaluate the calibration capability of the ultrasomics nomogram. Additionally, mapping DCA was utilized to evaluate the clinical utility of these predictive models.

### Statistical analysis

We compared the clinical and ultrasonic features of the participants utilizing an independent sample t-test, Mann-Whitney U test, or *X* ^2^ test, where appropriate. The threshold was set at a two-tailed p-value < 0.05, indicating statistical significance. **Table 1** shows the baseline clinical and ultrasonic features of the participants in the training and test cohorts respectively.

**Table 1:** Clinical and ultrasonic characteristics in the training and test cohorts.

Variable	Training cohort			Test cohort		
	Pancreatic cancer	PNET	P-value	Pancreatic Cancer	PNET	P-value
Age	60.09 ± 8.78	47.18 ± 13.64	<0.001	57.58 ± 10.55	46.23 ± 12.22	<0.001
Maximum diameter	37.19 ± 12.84	20.02 ± 12.07	<0.001	37.42 ± 13.00	24.25 ± 15.43	<0.001
Gender			0.102			0.702
0	43(49.43)	47(63.51)		21(52.50)	18(60.00)	
1	44(50.57%)	27(36.49)		19(47.50)	12(40.00)	
Shape			<0.001			<0.001
0	64(73.56)	21(28.38)		35(87.50)	14(46.67)	
1	23(26.44)	53(71.62)		5(12.50)	16(53.33)	
Margin			<0.001			0.003
0	37(42.53)	7(9.46)		20(50.00)	4(13.33)	
1	50(57.47)	67(90.54)		20(50.00)	26(86.67)	
Echo			0.023			0.423
0	3(3.45)	11(14.86)		2(5.00)	4(13.33)	
1	84(96.55)	63(85.14)		38(95.00)	26(86.67)	
uniformity			<0.001			0.174
0	65(74.71)	27(36.49)		30(75.00)	17(56.67)	
1	22(25.29)	47(63.51)		10(25.00)	13(43.33)	
Calcification						0.206
0	82(94.25)	73(98.65)	0.294	36(90.00)	30(100.00)	
1	5(5.75)	1(1.35)		4(10.00)	0(0)	
Cystic areas			<0.001			0.256
0	62(71.26)	72(97.30)		29(72.50)	26(86.67)	
1	25(28.74)	2(2.70)		11(27.50)	4(13.33)	
Location			0.144			0.917
0	44(50.57)	28(37.84)		23(57.50)	16(53.33)	
1	43(49.43)	46(62.16)		17(42.50)	14(46.67)	

Gender: “0” means female, “1” means male; Shape: “0” means irregular shape, “1” means regular shape; Margin: “0” means unclear margin of lesion, “1” means clear margin of lesion; Echo: “0” means means not hypoechoic of lesion, “1” means hypoechoic of lesion; uniformity: “0” means nonuniformity of echo; “1” means uniformity of echo; Calcification: “0” means no calcification, “1” means calcification; Cystic areas: “0” means no cystic areas, “1” means cystic areas; Location: “0” means head and uncinate process of the pancreas, “1” means body and tail of the pancreas.

## Results

### Patient population and ultrasonic characteristics

A total of 231 patients (102 women, 129 men) with pancreatic tumors, comprising 127 patients with pancreatic cancer and 104 patients with PNET, were included in this study, including 161 patients in the training cohort and 70 patients in the testing cohort. The findings illustrated that there was no significant difference in the gender ratio between the patients in the training and test cohorts. All the details of the clinical and ultrasonic characteristics are delineated in **Table 1**. The outcomes of this study revealed significant differences in age, maximum diameter, shape, margin characteristics, echo characteristics, and the presence of cystic degeneration between pancreatic cancer patients and PNET patients in the training cohort. However, no significant differences were observed in terms of echo characteristics or uniformity of the echo in the test cohort. The results of univariate and multivariable logistic regression analyses indicated that age, maximum diameter, and shape independently predicted the presence of PNET.

**Figure 3:**
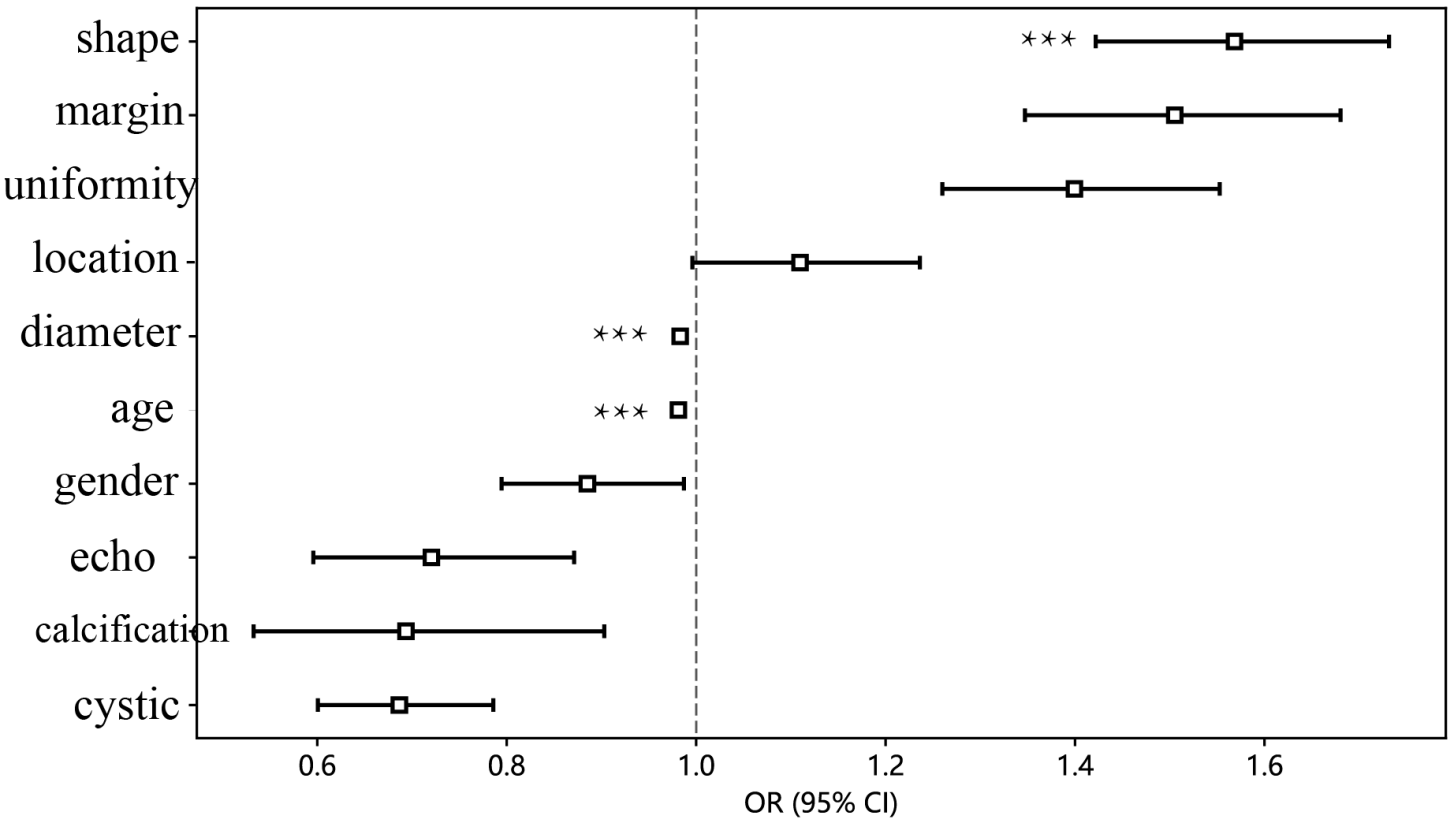
The forest map of univariate logistic regression of clinical and ultrasonic features. *** means P < 0.001.

The findings indicated that elderly individuals (OR 0.989; 95% CI 0.985 to 0.992), those with an enlarged maximum diameter (OR 0.990; 95% CI 0.987 to 0.993), and those with an irregular shape (OR 1.242; 95% CI 1.133 to 1.361) were more likely to be diagnosed with pancreatic cancer (**Table 2**; **Figure 3**).

**Table 2:** Univariate and multivariable logistic regression analyses for selecting clinical and ultrasonic features.

Variable	Univariate analysis		Multivariate analysis	
	OR(95% CI)	P-value	OR(95% CI)	P-value
Age	0.981(0.978, 0.985)	0.000	0.989(0.985,0.992)	0.000
Maximum diameter	0.983(0.980, 0.986)	0.000	0.990(0.987,0.993)	0.000
Shape	1.658(1.422,1.732)	0.000	1.242(1.133,1.361)	0.000
Margin	1.505(1.307,1.680)	0.000	1.124(1.017,1.242)	0.055
Echo	0.720(0.596, 0.871)	0.005	0.960(0.829,1.112)	0.646
uniformity	1.399(1.260,1.553)	0.000	1.057(0.967,1.156)	0.306
Calcification	0.693(0.533,0.903)	0.023	0.803(0.658,0.978)	0.068
Cystic areas	0.687(0.600, 0.786)	0.000	0.916(0.819,1.024)	0.197
Location	1.109(0.996,1.236)	0.115		
Gender	0.886(0.795,0.987)	0.066		

OR, odds ratio; CI, confidence interval.

### Ultrasomics feature extraction and screening

A comprehensive set of 7 categories and 107 manually derived ultrasomics features were obtained, comprising 18 first-order features, and 14 shape features, while the remaining features were texture features. Exhaustive information regarding these handcrafted features can be found in the **Supplementary Materials**.

**Figure 4:**
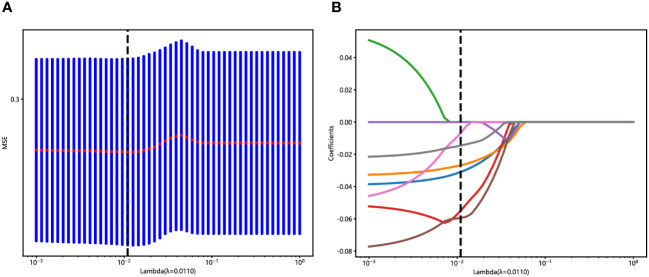
Ultrasomics feature selection with the LASSO regression model. **(A)** The LASSO model’s tuning parameter (λ) selection used 10-fold cross-validation via minimum criterion. The vertical lines illustrate the optimal value of the LASSO tuning parameter (λ). **(B)** LASSO coefficient profile plot with different log (λ) was displayed. The vertical dashed lines represent 6 ultrasomics features with nonzero coefficients selected with the optimal λ value.

An in-house feature analysis program, PyRadiomics, was utilized to extract all handcrafted features. **Supplementary Figure 2** presents the complete set of ultrasomics features along with their corresponding *p*-value results. Following the downscaling of ultrasomics features and LASSO logistic regression, a total of 6 ultrasomics features with nonzero coefficients were retained. The coefficients and mean standard errors (MSEs) of LASSO regression, resulting from the 10-fold validation, are depicted in **Figure 4**.

**Figure 5:**
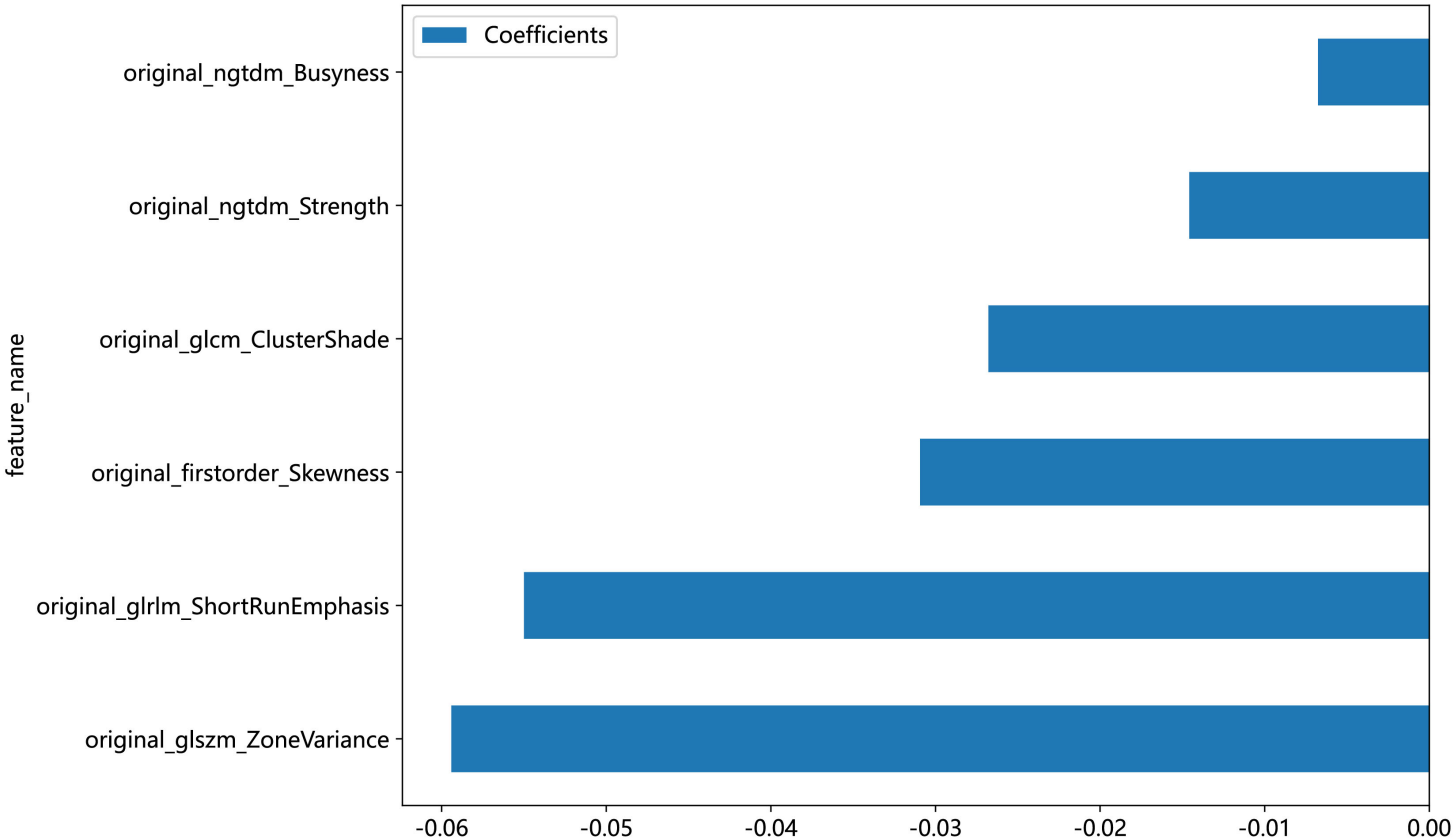
The bar graph of 6 ultrasomics features that achieved nonzero coefficients.

The coefficients of these retained 6 ultrasomics features are depicted in **Figure 5**, while the Ultrasomics score is shown as follows:

Ultrasomics score =0.45022 - 0.03094*original_firstorder_Skewness
-0.02680*original_glcm_ClusterShade
-0.05500*original_glrlm_ShortRunEmphasis
-0.05942*original_glszm_ZoneVariance
-0.00677*original_ngtdm_Busyness
-0.01459*original_ngtdm_Strength

### Ultrasomics signature and its performance

**Figure 6:**
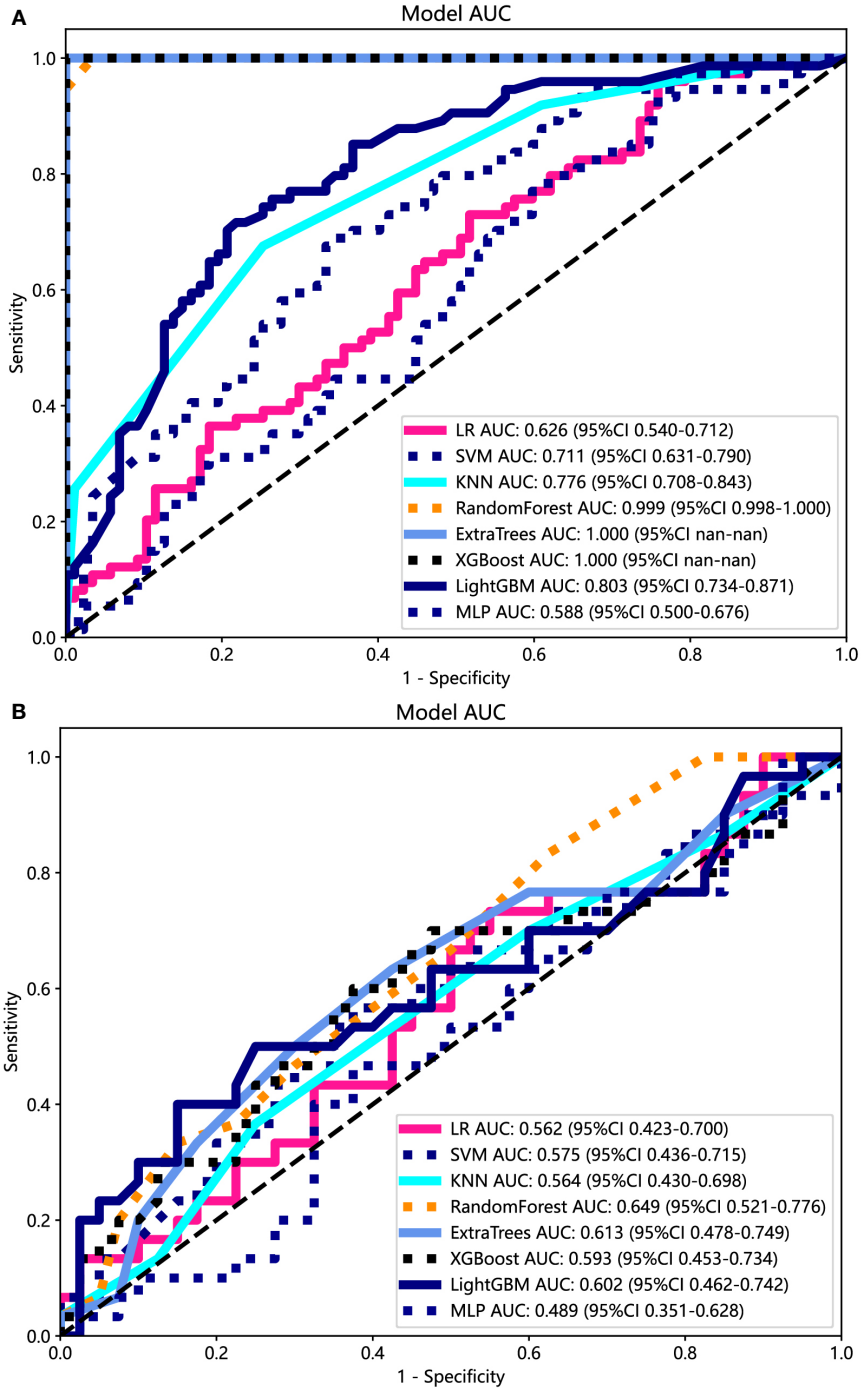
The ROC curves of different ultrasomics models based on eight machine-learning algorithms for predicting PNET. **(A)** The ROC curves of different ultrasomics models in the training cohort. **(B)** The ROC curves of different ultrasomics models in the test cohort.

The ROC curers and AUCs of the eight ultrasomics machine-learning models obtained with the eight mainstream machine-learning algorithms in the training and test cohorts are presented in **Figure 6**. Additionally, more details are shown in **Table 3**. Concerning the training cohort, the ExtraTrees model demonstrated superior performance, achieving an AUC of 1.000 and an accuracy of 1.000. Conversely, in the test cohort, the RF model emerged as the most effective ultrasomics model, exhibiting an AUC of 0.649, an accuracy of 0.600, a sensitivity of 0.567, a specificity of 0.775, a PPV of 0.550, and an NPV of 0.620.

**Table 3:** Diagnostic performance of different models for predicting PNET in training and test cohorts.

Model	Cohort	AUC(95% CI)	Accuracy	Sensitivity	Specificity	PPV	NPV
LR	Training	0.626(0.5400 - 0.7116)	0.571	0.432	0.690	0.542	0.588
	Test	0.562(0.4234 - 0.6999)	0.557	0.400	0.675	0.480	0.600
SVM	Training	0.711(0.6308 - 0.7903)	0.634	0.243	0.966	0.857	0.600
	Test	0.575(0.4362 - 0.7147)	0.586	0.133	0.925	0.571	0.587
KNN	Training	0.776(0.7080 - 0.8431)	0.714	0.676	0.747	0.694	0.612
	Test	0.564(0.4302 - 0.6982)	0.586	0.367	0.750	0.524	0.956
RF*	Training	0.999(0.9977 - 1.0000)	0.975	0.946	1.000	1.000	0.956
	Test	0.649(0.5215 - 0.7760)	0.600	0.567	0.775	0.550	0.620
ExtraTrees	Training	1.000(1.0000 - 1.0000)	1.000	1.000	1.000	1.000	1.000
	Test	0.613(0.4775 - 0.7491)	0.614	0.500	0.700	0.556	0.651
XGBoost	Training	1.000(1.0000 - 1.0000)	0.988	0.973	1.000	1.000	0.978
	Test	0.593(0.4530 - 0.7337)	0.586	0.500	0.650	0.517	0.634
LightGBM	Training	0.803(0.7342 - 0.8708)	0.720	0.581	0.839	1.000	1.000
	Test	0.602(0.4618 - 0.7423)	0.629	0.433	0.775	0.591	0.646
MLP	Training	0.588(0.4999 - 0.6756)	0.540	0.122	0.897	0.500	0.545
	Test	0.489(0.3508 - 0.6275)	0.557	0.033	0.950	0.333	0.567
Clinical signature*	Training	0.999(0.9961 - 1.0000)	0.988	0.986	0.989	0.986	0.989
	Test	0.844(0.7513 - 0.9370)	0.786	0.733	0.825	0.759	0.805
Radiomics nomogram*	Training	1.000(1.0000 - 1.0000)	1.000	1.000	1.000	1.000	1.000
	Test	0.884(0.8047 - 0.9635)	0.800	0.733	0.850	0.786	0.810

*Represents models were constructed based on RF.

LR, logistic regression; SVM, support vector machine; RF, random forest; KNN, k nearest neighbors; LightGBM, light gradient boosting machine; MLP, multilayer perceptron; XGBoost, extreme gradient boosting.

The ExtraTrees and XGBoost models presented a tendency toward overfitting in the training and test cohort. Contrarily, the RF model seemed to own better consistency between the training and test cohorts, with a great net benefit as presented in the DCA curve (**Supplementary Figure 3**). To establish the stability and sustainability of the ultrasomics machine-learning model, the RF model was determined to be the most suitable for subsequent analysis and defined as the ultrasomics signature model. Consequently, RF was selected as the foundational algorithm for developing the clinical-ultrasonic signature.

### Ultrasomics nomogram construction and assessment

**Figure 7:**
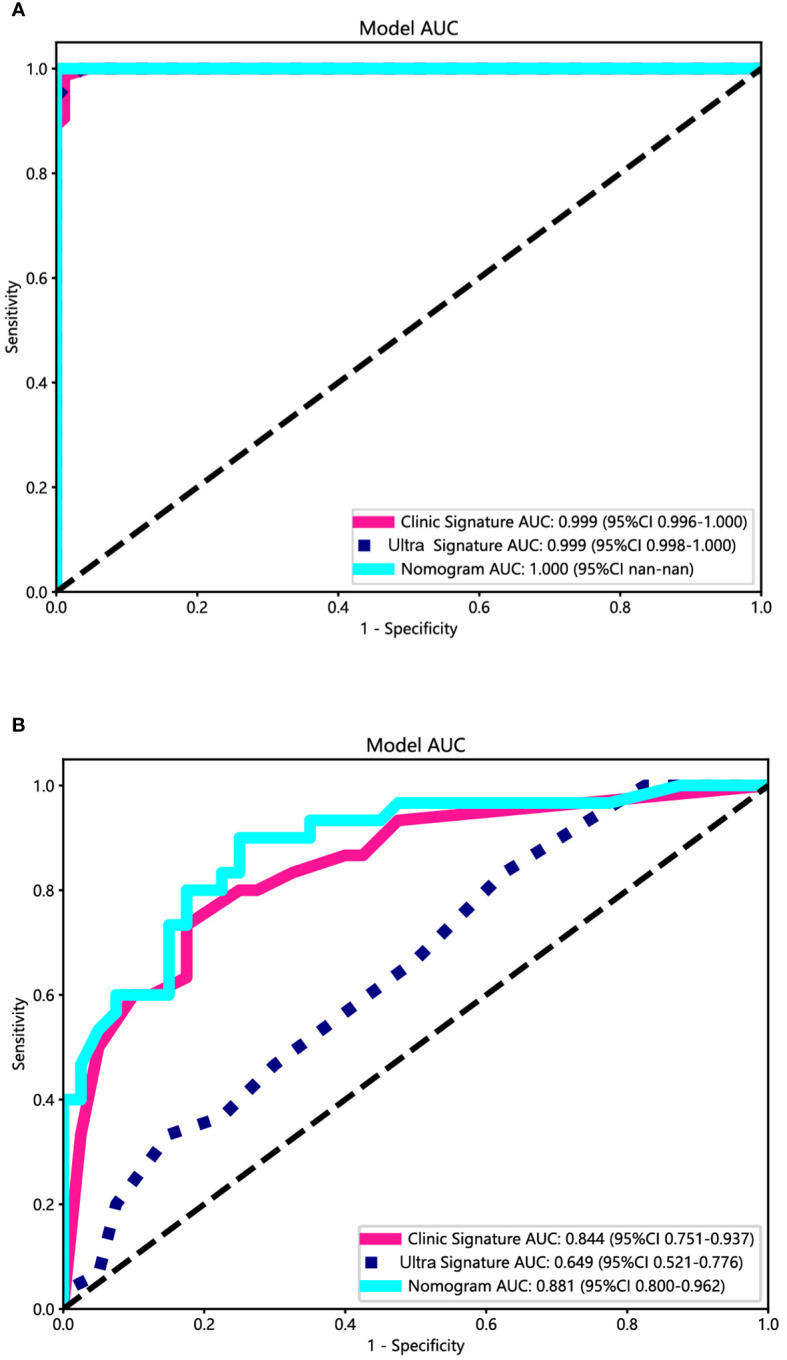
The ROC curves of the ultrasomics nomogram model (abbreviated “Nomogram”), clinical-ultrasonic signature (abbreviated “Clinic Signature”), and ultrasomics signature (abbreviated “Ultra Signature”) in the **(A)** training cohort and **(B)** test cohort, respectively.

**Figure 8:**
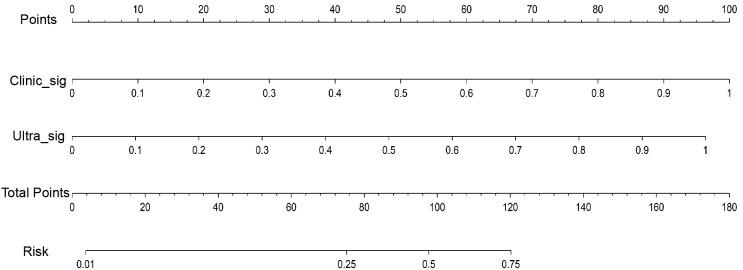
The ultrasomics nomogram model predicts PNET based on clinical-ultrasonic signature (abbreviated “Clinic_Sig”) and ultrasomics signatures (abbreviated “Ultra_Sig”) simultaneously. The nomogram is used by summing all points identified on the scale for each variable. The total points projected on the bottom scales indicate the probabilities of PNET.

Both the clinical-ultrasonic signature and ultrasomics signature appear to exhibit overfitting tendencies in the training cohort, as indicated in **Table 3**. Nevertheless, these two models demonstrated a good fit in the test cohort. To address this issue, an ultrasomics nomogram model was employed to combine the ultrasomics signature and clinical-ultrasonic signature, resulting in the most optimal performance achieved by constructing a more precise and consistent prediction model. The ROC curves of these three models, namely the clinical-ultrasonic signature model, ultrasomics signature model, and ultrasomics nomogram, are exhibited in **Figure 7** for both the training and test cohorts. To assess the effectiveness of these models, the Delong test was conducted. Fortunately, the AUC of the ultrasomics nomogram demonstrated significant superiority over the ultrasomics signature model and clinical-ultrasonic signature model in the testing cohort (**Table 4**), indicating that this ultrasomics nomogram (**Figure 8**) exhibited the highest level of diagnostic efficacy.

**Table 4:** The results of Delong test and Hosmer-Lemeshow test.

Model	P-value	
	Training cohort	Test cohort
Delong test		
Nomogram vs clinical signature	0.276	0.049
Nomogram vs ultrasomics signature	0.184	0.002
Hosmer-Lemeshow test		
ultrasomics signature	0.004	0.065
clinical signature	0.513	0.000
Nomogram	0.523	0.102

**Figure 9:**
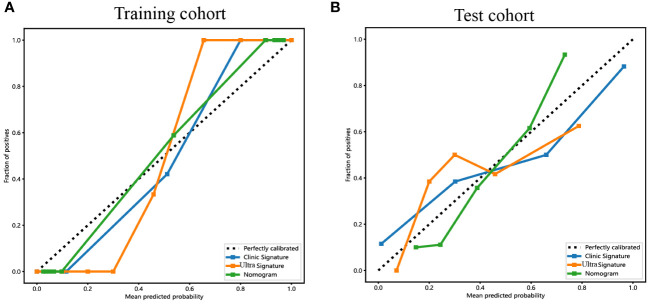
The calibration curves for the ultrasomics nomogram model (abbreviated “Nomogram”), clinical-ultrasonic signature (abbreviated “Clinic Signature”), and ultrasomics signature (abbreviated “Ultra Signature”) in the **(A)** training cohort and **(B)** test cohort, respectively.

The calibration curves of the ultrasomics nomogram performed respectable consistency between the predicted and observed PNET in both the training and test cohorts. The results of the H-L test illustrated that the ultrasomics nomogram had greater prediction accuracy than the other models (**Table 4**). The calibration curves for the training and test cohorts are displayed in **Figure 9**.

**Figure 10:**
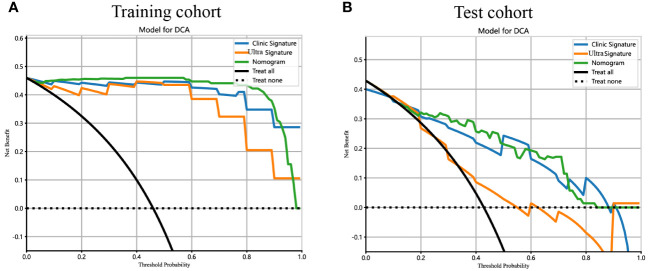
The DCA curves for the ultrasomics nomogram model (abbreviated “Nomogram”), clinical-ultrasonic signature (abbreviated “Clinic Signature”), and ultrasomics signature (abbreviated “Ultra Signature”) in the **(A)** training cohort and **(B)** test cohort, respectively.

Additionally, DCA curves were conducted to assess the performance of each model, and the results are depicted in **Figure 10**. The ultrasomics nomogram demonstrated a significant net benefit for patients receiving intervention based on its prediction probability compared to the hypothetical scenarios where no prediction model was accessible, such as the treat-all or treat-none schemes. Furthermore, the ultrasomics nomogram exhibited elevated values compared to those of the other signatures in both the training and test cohorts. Consequently, this ultrasomics nomogram has been shown to potentially enhance the clinical efficacy of predicting PNET, before surgery and EUS-FNA procedures.

## Discussion

The ability of EUS to perform comprehensive ultrasound scans of the entire pancreas from the stomach and duodenum, while maintaining proximity and minimal interference, has been widely acknowledged. This capability enables the generation of high-resolution images and facilitates the visualization of intricate anatomical features. Furthermore, the potential for tissue acquisition through EUS-FNA enhances the diagnostic accuracy of EUS, positioning it as one of the most reliable approaches for diagnosing PNET. Moreover, endoscopic ultrasound-fine needle aspiration/biopsy (EUS-FNA/B) is a precise method for determining the pathological grading of PNET, with a pooled estimate rate for overall concordance of 80.3% (95% CI 75.6–85.1) (17). Based on a comprehensive analysis of ten prior studies involving a total of 261 participants, EUS had been shown to demonstrate a commendable average predictive accuracy of 90% (with a range of 77–100%) in the diagnosis of PNET (18). The Chinese Neuroendocrine Tumor (CSNET) collaboration has reached a consensus on the treatment strategy for scattered solitary small NF-PNET, recommending surgical resection and lymph node dissection for most <2cm NF-PNET (19). According to Manta et al, patients diagnosed with pathologically confirmed PNET underwent simultaneous preoperative CT and EUS examinations. The overall detection rate of CT for detecting lesions was 64%. However, for lesions with a diameter less than 10 mm and a range of 11–20 mm, the CT missed diagnosis rate was remarkably high at 68% and 15% respectively. Conversely, EUS had demonstrated a successful detection rate of approximately 47.5% for small pancreatic PNET lesions measuring between 4 and 10 mm in diameter (20). Preoperative EUS imaging for F-PNET can accurately assess the correlation and proximity of the lesion to the main pancreatic duct, thereby playing a pivotal role in determining the appropriate surgical approach, whether it is local or radical resection (21). In some PNET patients, MEN1 may be complicated, and in these patients, multiple small pancreatic lesions are common. Given the limited efficacy of conventional CT and MRI for identifying these small lesions, the utilization of EUS and contrast-enhanced EUS is highly recommended (22). Consequently, EUS plays a crucial role in the detection, diagnosis, and operation strategy selection of PNET.

As previously elucidated, our study provides evidence that patients with PNET may exhibit a younger age profile, and the lesions are more likely to have a shorter diameter and regular shape, than those with pancreatic cancer. Furthermore, the variables of age, maximum diameter, and shape were found to be independent predictors of PNET. In line with our findings, Iordache S observed a statistically significant difference in the age of patients with pancreatic adenocarcinoma compared to those with PNET (62.40 ± 11.24 vs 62.40 ± 11.24, P = 0.0119) (23). PNET was commonly distinguished by low-intensity echoes, well-defined borders, regular roundness in shape, vascularization, and uniform internal echo patterns (24). Furthermore, it has been reported that the median volume of PNET was notably smaller than that of pancreatic adenocarcinomas (25). In summary, the characteristics of the clinical and EUS ultrasonic features observed in this study were extremely consistent with those of previous reports in the literature, further validating the reliability of the research results.

The acquisition of technical expertise via EUS was a demanding endeavor, characterized by a steep and intricate learning curve (26). According to the American Society for Gastrointestinal Endoscopy (ASGE), a comprehensive EUS practice should encompass a minimum of two years of routine gastrointestinal endoscopy experience, coupled with at least one year of specialized training in biliopancreatic EUS (27). Contrary to the normal US and digestive endoscopy, EUS necessitated a higher level of proficiency in endoscopic techniques, exceptional spatial perception, extensive diagnostic experience, and a comprehensive understanding of anatomical knowledge by physicians. These factors contributed to the variability in recognizing and interpreting macroscopic imaging characteristics of EUS images, thereby limiting the specificity and sensitivity of EUS diagnosis (28). Cystic changes were observed in 8–17% of PNET patients and manifest as unilocular, septated, microcystic, or mixed solid and cystic formations. Nevertheless, these cystic alterations lacked specificity, rendering differentiation through radiology or EUS alone a challenging task (29). The subjective interpretation of EUS image features, including tumor size, shape, border, and vessel invasion, relied heavily on the operator’s experience, resulting in inadequate homogeneity. Therefore, there is a pressing need for a novel approach utilizing EUS imaging that offers enhanced objectivity and accuracy in predicting PNET.

Radiomics enables the extraction of multidimensional data from medical images that surpass human visual assessment. By converting medical images into extractable data, this approach facilitates the design of classification models utilizing machine learning algorithms. Consequently, this approach enhanced the identification of various tumor types with increased reliability and objectivity (6, 30). Recently, the academic community has shown increasing interest in the exploration of EUS-based ultrasomics. In a multicenter study, Li XY demonstrated that the integration of machine learning algorithms with EUS ultrasomics features enabled the development of an effective classification model for assessing the malignancy grade of gastrointestinal stromal tumors (GISTs) (6). Gu et al. successfully devised a deep-learning ultrasomics model utilizing EUS images to diagnose pancreatic ductal adenocarcinoma. This model effectively mitigated the diagnostic disparity among ultrasound endoscopy physicians of varying expertise levels, thereby enhancing the precision of their diagnoses (31). The EUS-based ultrasomics model was developed to accurately distinguish between gastric GISTs, smooth muscle tumors, and nerve sheath tumors (32). Regrettably, the literature lacked published studies that have utilized EUS imaging ultrasomics to diagnose and predict PNET.

Our study revealed that a comprehensive set of 107 EUS imaging ultrasomics features were initially extracted. Subsequently, through rigorous statistical analyses including variance analysis, correlation analysis, and LASSO regression analysis, a subset of 6 ultrasomics features were identified as highly significant and definitively associated with PNET. Multiple mainstream machine learning algorithms were used simultaneously to construct the most appropriate two-class prediction model for distinguishing PNET from pancreatic cancer, to overcome the limitations of single algorithms. In this instance, the RF algorithm seemed to demonstrate superior accuracy and consistency and was subsequently applied for further model development. In the training cohort, both the ultrasomics signature model and clinical-ultrasonic signature model based on the RF algorithm achieved respectable AUCs. However, the effectiveness of the ultrasomics signature model was inferior to that of the clinical-ultrasonic signature model. Interestingly, we constructed an ultrasomics nomogram by combining the ultrasomics signature and clinical-ultrasonic signature as described previously, with a significant improvement in prediction efficiency in the testing cohort. Moreover, the DeLong test and H-L test have confirmed the validity and accuracy of this ultrasomics nomogram.

The RF algorithm, an ensemble learning technique that integrates a variety of decision trees, stands out for its exceptional accuracy compared to other existing algorithms and has the potential to enhance selection during classification prediction (33). Our results illustrated that although the clinical-ultrasonic signature model owned relatively high effectiveness, the ultrasomics nomogram achieved better predictive performance. To the best of our knowledge, this study represents the initial demonstration that the EUS-based ultrasomics nomogram significantly and efficiently improved the prediction of PNET. Given the higher detection rate of PNET and the superior preoperative lesion detail assessment capabilities of EUS compared to CT and MRI, these findings are expected to contribute to the advancement of EUS utilization in the management of PNET.

Despite the notable efficacy of the ultrasomics nomogram utilizing EUS imaging, this study is subject to certain limitations. Notably, this retrospective analysis was conducted within a single institution based on different EUS devices, potentially introducing selection and systematic bias. Additionally, all boundary definitions were derived from manual segmentation in image segmentation, and bias was inevitable (34). Furthermore, it is essential to acknowledge that this study utilized only conventional EUS images, disregarding the potential added value of contrast-enhanced EUS and elastography EUS techniques (22, 35, 36). Hence, it is imperative to undertake future studies on EUS-based ultrasomics for PNET that encompass multiple centers, large sample sizes, prospective designs, and multimodal approaches based on the same equipment. Another limitation of this study was that all the ultrasomics features were assessed in two instead of three dimensions. We expect that extending this work to three dimensions based on EUS videos may yield improved model accuracy and representability in the future. Moreover, regarding the critical value of pathological grade and tumor size in the diagnosis, treatment, and prognosis of PNET (37), the predictive value of EUS-based ultrasomics for PNET pathological grading and small tumors (<15mm) also needs to be further evaluated. A concurrent EUS-FNA subgroup analysis to further clarify the accuracy of EUS-based ultrasomics would also be of high value. Additionally, in clinical practice, the artifacts and noise of EUS images are not always avoided, so integrating deep learning techniques based on automatic image segmentation could mitigate bias and enhance the generalizability and applicability of the models.

## Conclusion

In summary, an efficient EUS-based ultrasomics nomogram, incorporating clinical-ultrasonic and ultrasomics signatures, was proposed and validated for predicting pancreatic cancer and PNET. These outcomes provide potential novel insight into improving clinical prediction and treatment strategies for PNET.

## Data availability statement

The original contributions presented in the study are included in the article/**Supplementary Material**. Further inquiries can be directed to the corresponding authors.

## Ethics statement

The studies involving humans were approved by the Medical Ethics Committee of The First Affiliated Hospital of Guangxi Medical University (No. 2023-E732-01). The studies were conducted in accordance with the local legislation and institutional requirements. The participants provided their written informed consent to participate in this study. Written informed consent was obtained from the individual(s) for the publication of any potentially identifiable images or data included in this article.

## Author contributions

SM: Conceptualization, Data curation, Formal analysis, Funding acquisition, Investigation, Methodology, Project administration, Resources, Software, Supervision, Validation, Visualization, Writing – original draft, Writing – review & editing. CH: Formal analysis, Software, Visualization, Writing – original draft, Writing – review & editing. YW: Writing – original draft, Writing – review & editing. HZ: Formal analysis, Investigation, Methodology, Resources, Writing – original draft. HW: Writing – original draft, Writing – review & editing. HQ: Writing – original draft, Writing – review & editing. HJ: Funding acquisition, Methodology, Resources, Writing – original draft, Writing – review & editing. SQ: Conceptualization, Data curation, Formal analysis, Funding acquisition, Investigation, Methodology, Project administration, Resources, Software, Supervision, Validation, Visualization, Writing – original draft, Writing – review & editing.

## Supplementary material

Supplementary Figure 1 The correlation coefficient matrix of ultrasomics features.

Supplementary Figure 2 The violin plot of each ultrasomics feature along with their corresponding p-value results.

Supplementary Figure 3 The DCA curves of RF ultrasomics models in the training **(A)** and test **(B)** cohorts.

## Glossary

**Table:**

EUS	endoscopic ultrasound
PNET	pancreatic neuroendocrine tumors
LASSO	least absolute shrinkage, and selection operator
LR	logistic regression
SVM	support vector machine
RF	random forest
KNN	k nearest neighbors
LightGBM	light gradient boosting machine
MLP	multilayer perceptron
XGBoost	extreme gradient boosting
ROC	receiver operator characteristic
AUC	the area under the curve
OR	odds ratio
CI	confidence interval
DCA	decision curve analysis
F-PNET	functional pancreatic neuroendocrine tumors
NF-PNET	non-functional pancreatic neuroendocrine tumors
CT	computed tomography
MRI	magnet resonance imaging
US	ultrasonography
ENETS	European Neuroendocrine Tumor Society
CAD	computer-aided detection
AI	artificial intelligence
EUS-FNA	EUS-guided fine-needle aspiration
PACS	Picture Archive and Communication System
DICOM	Digital Imaging and Communications in Medicine
ICC	intra-class correlation coefficient
ROI	region of interest
GLCM	Gray Level Co-occurrence Matrix
GLRLM	Gray Level Run Length Matrix
GLSZM	Gray Level Size Zone Matrix
NGTDM	Neighborhood Gray-level Difference Matrix
IBSI	image biomarker standardization initiative
PPV	positive predictive value
NPV	negative predictive value
CSNET	Chinese Neuroendocrine Tumor
ASGE	American Society for Gastrointestinal Endoscopy
GISTs	gastrointestinal stromal tumors
