# Supplementary figures and images for: Construction and validation of an endoscopic ultrasonography-based ultrasomics nomogram for differentiating pancreatic neuroendocrine tumors from pancreatic cancer
Source: Front Oncol. 2024 May 23;14:1359364. doi: 10.3389/fonc.2024.1359364 (PMC11158619; doi:10.3389/fonc.2024.1359364)

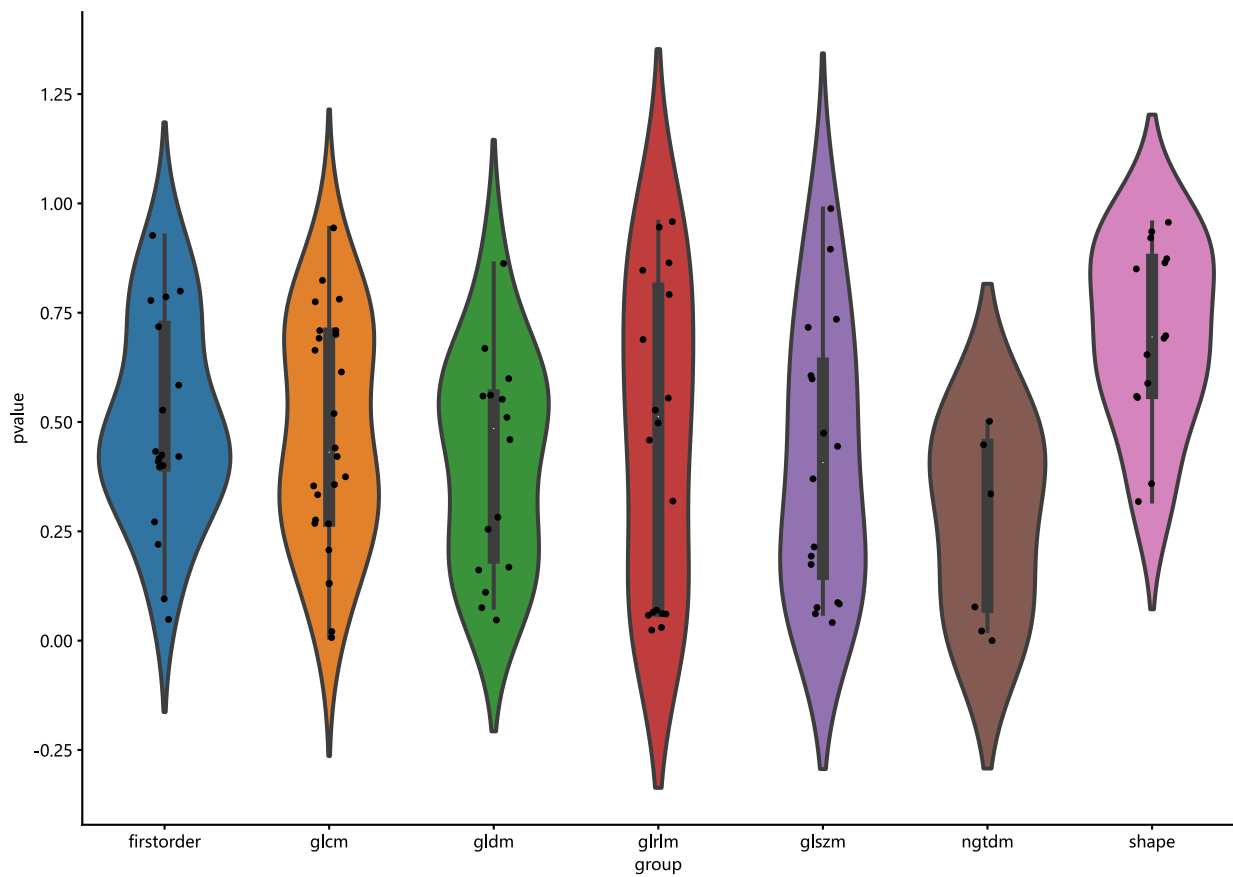

Supplement: Supplementary Figure 2 — The violin plot of each ultrasomics feature along with their corresponding p-value results. [file DataSheet_2.pdf]

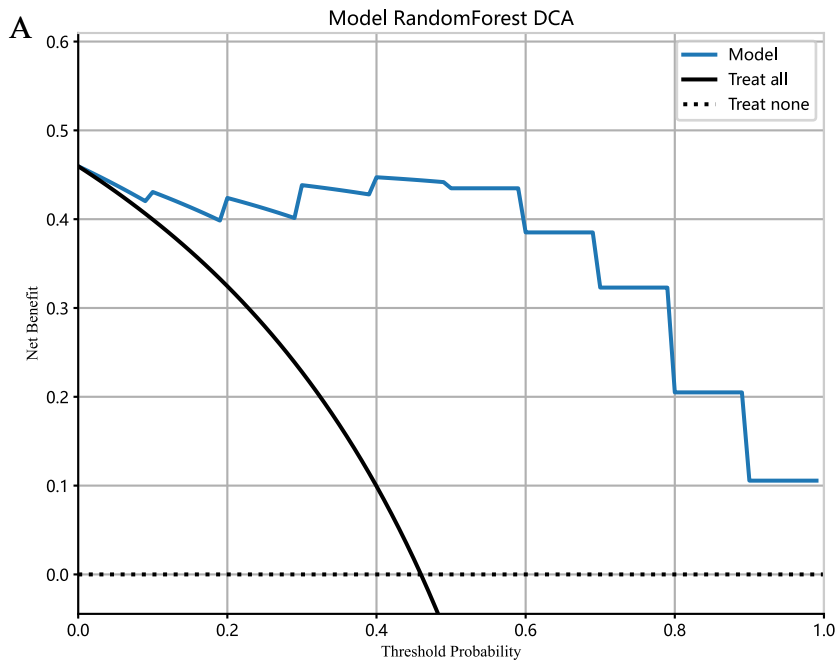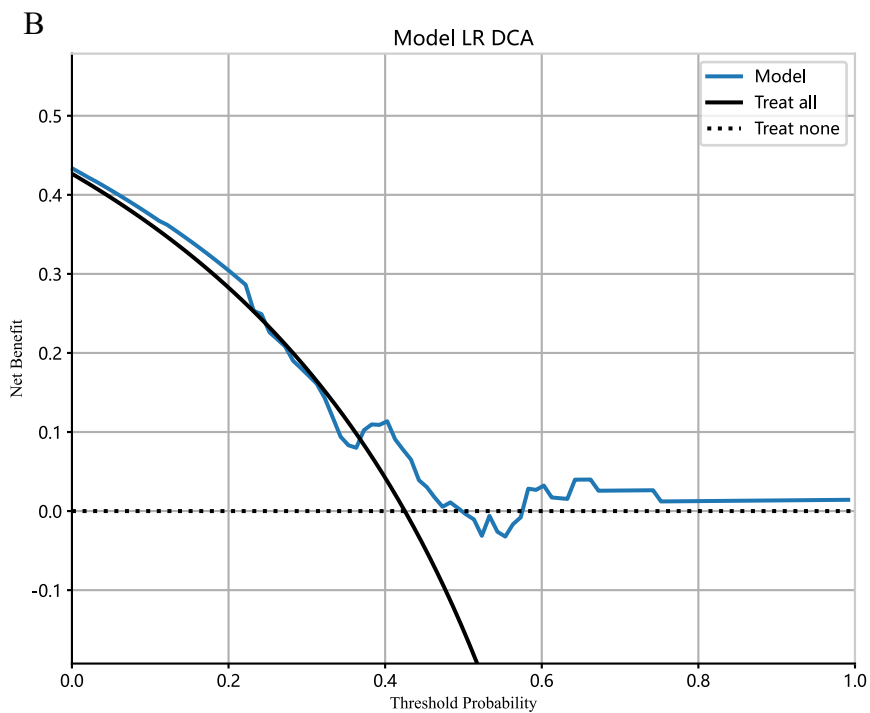

Supplement: Supplementary Figure 3 — The DCA curves of RF ultrasomics models in the training (A) and test (B) cohorts. [file DataSheet_3.pdf]
