# Supplementary material for: Construction and validation of an endoscopic ultrasonography-based ultrasomics nomogram for differentiating pancreatic neuroendocrine tumors from pancreatic cancer
Source: Front Oncol. 2024 May 23;14:1359364. doi: 10.3389/fonc.2024.1359364 (PMC11158619; doi:10.3389/fonc.2024.1359364)
Supplement: Supplementary Figure 1 — The correlation coefficient matrix of ultrasomics features. [file DataSheet_1.pdf]

	original_firstorder_Skewness	original_glcm_ClusterShade	original_gldm_SmallDependenceEmphasis	original_glrlm_LongRunEmphasis	original_glrlm_RunLengthNonUniformityNormalized	original_glrlm_RunPercentage	original_glrlm_RunVariance	original_glrlm_ShortRunEmphasis	original_glszm_LargeAreaEmphasis	original_glszm_LargeAreaLowGrayLevelEmphasis	original_glszm_ZonePercentage	original_glszm_ZoneVariance	original_ngtdm_Busyness	original_ngtdm_Strength
original_firstorder_Skewness	1.000	0.220	0.121	−0.148	0.160	0.161	−0.147	0.157	−0.109	−0.100	0.133	−0.109	−0.128	0.156
original_glcm_ClusterShade	0.220	1.000	0.182	−0.125	0.193	0.179	−0.112	0.189	−0.050	−0.054	0.179	−0.050	−0.087	0.235
original_gldm_SmallDependenceEmphasis	0.121	0.182	1.000	−0.511	0.729	0.709	−0.481	0.694	−0.218	−0.233	0.992	−0.217	−0.405	0.730
original_glrlm_LongRunEmphasis	−0.148	−0.125	−0.511	1.000	−0.798	−0.852	0.995	−0.861	0.734	0.792	−0.520	0.733	0.814	−0.245
original_glrlm_RunLengthNonUniformityNormalized	0.160	0.193	0.729	−0.798	1.000	0.991	−0.753	0.987	−0.438	−0.463	0.747	−0.437	−0.583	0.397
original_glrlm_RunPercentage	0.161	0.179	0.709	−0.852	0.991	1.000	−0.812	0.995	−0.482	−0.511	0.727	−0.481	−0.633	0.376
original_glrlm_RunVariance	−0.147	−0.112	−0.481	0.995	−0.753	−0.812	1.000	−0.816	0.756	0.815	−0.490	0.755	0.826	−0.228
original_glrlm_ShortRunEmphasis	0.157	0.189	0.694	−0.861	0.987	0.995	−0.816	1.000	−0.489	−0.521	0.709	−0.488	−0.635	0.365
original_glszm_LargeAreaEmphasis	−0.109	−0.050	−0.218	0.734	−0.438	−0.482	0.756	−0.489	1.000	0.929	−0.218	1.000	0.794	−0.095
original_glszm_LargeAreaLowGrayLevelEmphasis	−0.100	−0.054	−0.233	0.792	−0.463	−0.511	0.815	−0.521	0.929	1.000	−0.233	0.928	0.826	−0.101
original_glszm_ZonePercentage	0.133	0.179	0.992	−0.520	0.747	0.727	−0.490	0.709	−0.218	−0.233	1.000	−0.217	−0.413	0.716
original_glszm_ZoneVariance	−0.109	−0.050	−0.217	0.733	−0.437	−0.481	0.755	−0.488	1.000	0.928	−0.217	1.000	0.793	−0.094
original_ngtdm_Busyness	−0.128	−0.087	−0.405	0.814	−0.583	−0.633	0.826	−0.635	0.794	0.826	−0.413	0.793	1.000	−0.214
original_ngtdm_Strength	0.156	0.235	0.730	−0.245	0.397	0.376	−0.228	0.365	−0.095	−0.101	0.716	−0.094	−0.214	1.000
